# Identification of Novel Extracellular-Signal-Regulated Kinase 2 Inhibitors Through Machine Learning-Driven De Novo Design, Molecular Docking, and Free-Energy Perturbation

**DOI:** 10.3390/ph19020337

**Published:** 2026-02-20

**Authors:** Ibrahim A. Alsarra, Mahima Sudhir Kolpe, Md Ataul Islam

**Affiliations:** 1 Department of Pharmaceutics and Industrial Pharmacy, College of Pharmacy, King Saud University, Riyadh 11451, Saudi Arabia; ialsarra@ksu.edu.sa; 2 SilicoScientia Private Limited, Nagananda Commercial Complex, No. 07/3, 15/1, 18th Main Road, Jayanagar 9th Block, Bengaluru 560041, India; mahima.kolpe@silicoscientia.com; 3 SilicoScientia Private Limited, Centre for Cellular and Molecular Platforms (C-CAMP), GKVK Campus, Bellary Road, Bengaluru 560065, India

**Keywords:** ATP-binding site, de novo drug design, ERK2, free-energy perturbation, molecular docking, molecular dynamics simulation

## Abstract

**Background**: The extracellular-signal-regulated kinase (ERK) cascade regulates cell proliferation, differentiation, and survival, and ERK2 mediates substrate phosphorylation, influencing gene expression and cellular functions. **Methods**: In the current study, a pool of new molecules was generated using the DeLA-Drug, a machine learning (ML)-assisted de novo design tool. The chemical space was reduced through a similarity search against active ERK2 inhibitors and molecular docking with AutoDock vina, followed by pharmacokinetic assessment in DeepPK. Poses of the final selected molecules were refined in DiffDock, and dynamicity was assessed through molecular dynamics (MD) simulation. Finally, the free-energy perturbation (FEP)-based binding affinity was explored in Gromacs2023.4. **Results**: From the above approaches, four molecules (Ek1, Ek2, Ek3, and Ek4) were identified as promising candidates with favorable binding interactions. Molecular docking revealed that the selected molecules exhibited higher binding affinity for ERK2, ranging from −9.50 to −10.50 kcal/mol. The dynamics assessment via MD simulation clearly revealed their strong association with ERK2, corroborated by the lower deviation of the ERK2 backbone in dynamic states. All four screened molecules have satisfactory pharmacokinetic properties, medicinal chemistry properties, and good synthetic accessibility scores, indicating their potential as drug-like compounds under Lipinski’s rule of five to inhibit or modulate ERK2 activity. The FEP energy of Ek1 was found to be −26.85 kJ/mol, which is higher than the standard molecule (−22.77 kJ/mol) and indicates its strong affinity toward ERK2. **Conclusions**: These results suggest that all proposed ERK2 modulators are potential avenues for future drug discovery targeting ERK2, subject to experimental validation.

## 1. Introduction

The Ras (Rat sarcoma)/Raf (Rapidly Accelerated Fibrosarcoma)/MEK (Mitogen-activated protein kinase)/ERK (extracellular-signal-regulated kinase) signaling pathway is crucial for transmitting extracellular signals to regulate gene expression and control various cellular protein functions [1,2]. This cascade of proteins, including Raf, MEK, and ERK, is essential for many fundamental cellular functions such as proliferation, differentiation, and survival [1]. ERK proteins, especially ERK2, are vital because they activate many targets inside the cell [3,4]. These targets include transcription factors that regulate the production of various proteins involved in cell cycle control and apoptosis [5,6,7]. Additionally, ERK2 is notable for its ability to phosphorylate a wide range of targets across different cellular compartments. Such widespread influence positions ERK2 as a key component in the pathway, making it a promising target for treatment in diseases marked by abnormal cell growth and survival. Given its critical role in these processes, ERK2 emerged as a promising target for the development of drugs to treat diseases such as cancer and inflammatory disorders. Although drugs targeting this pathway effectively treat cancers, they are limited by side effects and resistance [1].

Structurally, ERK2 adopts the typical kinase fold, consisting of an N- and a C-lobe connected by a flexible hinge region [2]. Such architecture is common among most protein kinases and facilitates substrate binding and catalysis. ATP binds between the lobes in the deep groove (ATP site) [3], whose architecture creates a deep cleft, the ATP-binding site, where adenosine triphosphate (ATP) binds to fuel ERK2’s enzymatic activity. The smaller N-terminal lobe contains a five-stranded antiparallel beta-sheet (β1–β5) crucial for ATP binding. Specific strands within this sheet interact with the adenine base of ATP. On the other hand, the C-terminal lobe is a bit larger and consists of various alpha helices and additional beta sheets [4]. This lobe includes the catalytic domain, which contains key amino acid residues responsible for protein kinase activity. Other notable secondary structures include a glycine-rich loop and an activation segment, both of which play a role in ERK2 function [4]. ERK2 holds substantial clinical relevance as a central driver of proliferation and survival in RAS/MAPK-hyperactive cancers, including melanoma, colorectal, lung, and pancreatic carcinomas, while simultaneously mediating pro-inflammatory cytokine production and immune cell activation in chronic inflammatory and autoimmune disorders [5]. Aberrant activation of the ERK pathway has been implicated in the pathogenesis of various diseases, including cancer, inflammatory disorders, and neurological conditions [6,7]. Targeting the ATP-binding site of ERK2 theoretically disrupts its enzymatic activity and hinders the downstream signaling responsible for disease progression [8,9,10]. This approach may offer several advantages. First, targeting the ATP-binding site is a well-established strategy for kinase inhibition, with many successful inhibitors and drugs already in use [11,12,13]. Second, the ATP-binding site is highly conserved across kinases, which could enable the development of selective inhibitors with minimal off-target effects.

Several ERK1/2 inhibitors, such as SCH772984 [14], FR180204 [15], BVD-523 [16], and others, have shown promise in preclinical and early clinical studies but face significant limitations that hinder their widespread clinical use. Reports indicate that no ERK1/2 inhibitors have received FDA approval as of 2025, mainly due to the rapid development of resistance mechanisms, such as BRAF amplification, MEK1/2 mutations, and ERK feedback reactivation, which reduce their effectiveness in MAPK-driven cancers [17]. Traditional ATP-competitive inhibitors like SCH772984 and FR180204 face issues such as limited selectivity, high toxicity, poor oral bioavailability, high molecular weight, and suboptimal pharmacokinetics, which have contributed to clinical failures and narrow therapeutic windows [18]. These challenges emphasize the need for new research to develop advanced ERK2 inhibitors with better drug-like properties, including increased ligand efficiency, enhanced lipophilicity, and improved plasma stability, to maximize tumor targeting and minimize systemic toxicity.

A computationally exhaustive de novo compound design approach enables exploration of a wider chemical space beyond known drugs, potentially leading to entirely new therapeutic solutions. Therefore, the de novo drug design surpasses the limits of traditional methods by creating completely new candidate molecules based on specified chemical properties and biological targets [19]. DeLA-Drug (Deep Learning Algorithm for Automated Design of Drug-like Analogues) [20] is a valuable tool for de novo compound design, mainly focused on generating analogues of existing molecules. Utilizing the recurrent neural network (RNN) model, DeLA-Drug employs a strategy called “Sampling with Substitutions” (SWS) [20]. It analyzes the query compound’s SMILES string and strategically suggests modifications at specific locations within the molecule.

Henceforth, by creating a library of de novo-designed molecules specifically aimed at targeting the ATP-binding site of ERK2, we sought to explore this uncharted chemical space, guided by a known set of ERK2 inhibitors. Subsequently, DiffDock [21], another AI/ML algorithm, was used to identify the key site. Moreover, estimating pharmacokinetic and toxicity profiles with synthetic accessibility efficiently predicts the liability of the deciphered drug candidate. A long-range all-atomistic molecular dynamics (MD) simulation, coupled with binding free-energy calculations using the MM-GBSA (Molecular Mechanics Generalized Born Surface Area) [22] approach, was employed to identify four potential ERK2 inhibitors/modulators. Furthermore, free-energy perturbation (FEP) [23] was used to estimate the binding energy of the molecules. Innovative ERK2 inhibitors were developed using generative AI, then validated through physics-based molecular docking and high-accuracy FEP calculations. This work yields molecules that are not only potent but also have a higher chance of experimental success compared to conventionally designed or purely AI-generated candidates. These findings suggest promising prospects for future drug discovery endeavours targeting the ATP site of ERK2.

## 2. Results

**Figure 1:**
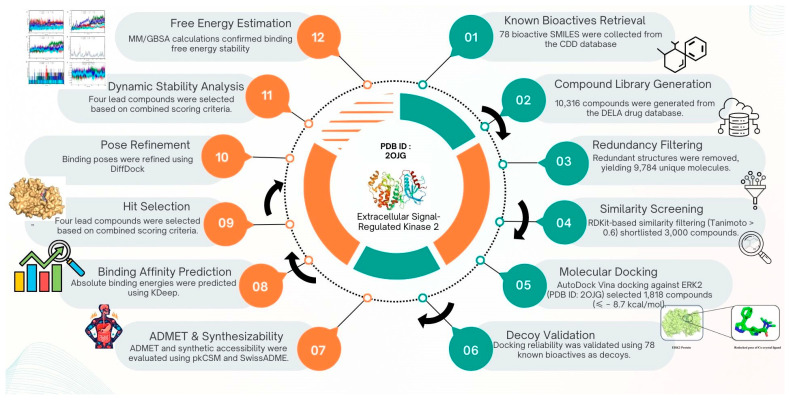
Workflow representing the key steps followed in the present study for the identification of ERK2 protein modulators.

De novo drug design is a crucial approach for developing new bioactive molecules that can target dysregulated MAPK/ERK signaling in cancer and inflammation-related diseases. An advanced DeLA-Drug tool [20] was used to generate inhibitor compounds targeting ERK2. The stepwise approach involved identifying the active sites of the ERK2 protein and focusing on these specific regions. Subsequently, a dataset comprising 298 bioactive compounds was curated from an extensive literature review, and 78 active compounds were selected based on their K_i_ values. The above set was docked, and the binding affinity was recorded (Supplementary data, Table S1). It was found that all 78 of the mentioned molecules possess a strong affinity for the ERK2 protein. Utilizing these 78 active molecules as input for a deep-learning-based tool, DeLA-Drug, 10,316 new molecules were generated. Deploying the ‘RDKit’ [24] module for fingerprint-based similarity search against the 78 active molecules, 3000 molecules had a Tanimoto coefficient of 0.6 or above. The 3000 new compounds were subjected to molecular docking studies. This molecular coordination study identified 1818 molecules with higher binding affinity than the standard molecule with PubChem ID: 91899270 (PubChem91899270). Pharmacokinetic analysis of these compounds identified 26 potential candidates. These 26 compounds were further evaluated for toxicity, and the top 4 were selected based on protein–ligand interactions, binding energy, and structural diversity. These four selected molecules exhibit promising characteristics and are deemed strong candidates for targeting the active sites of the ERK2 protein. The stepwise workflow of the entire pipeline is given in Figure 1. Detailed insights into each stage of this process are elaborated upon in subsequent sections.

### 2.1. Selection of ERK2 Protein and Active Site Identification

**Figure 2:**
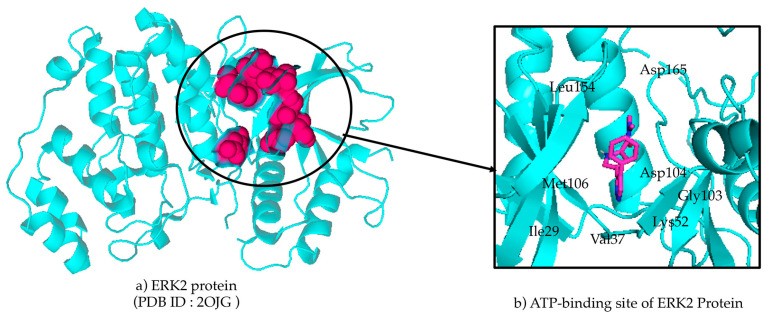
(**a**) The secondary structure of protein represents the active site in red CPK form, and (**b**) the ligand (represented in sticks) shows the orientation surrounded by the active site residues.

The structural evaluation of the protein of interest, ERK2, was performed in PyMol [25]. ERK2 is known for its role in ATP-dependent phosphorylation of various substrates, including downstream kinases and transcription regulators [26]. The protein structure with 2OJG was retrieved from the RCSB-Protein Data Bank (PDB) [27], and the ATP-binding active site is shown in Figure 2.

### 2.2. Setting Up Small Molecular Candidates for ERK2 Inhibition

The molecules generated by DeLA-Drug were used for a similarity search against the active set of 78 molecules. The similarity search aimed to retain de novo-designed molecules that are similar to known ERK2-active molecules. The fingerprint-based similarity search was performed using the RDKit. The molecules were ranked based on the Tanimoto coefficient, and compounds with a high similarity score (≥0.60) were retained. Based on the above screening criteria, a total of 3000 molecules were similar to the ERK2 active molecules. It is obvious that similar molecules possess similar characteristics; hence, the above molecules were found to exhibit high structural similarity to known ERK2 active compounds. Therefore, it would not be wrong to say that the above molecules might have comparable characteristics to ERK2 inhibitors or that a few molecules behave better than the existing ones.

### 2.3. Molecular Docking Analysis

**Figure 3:**
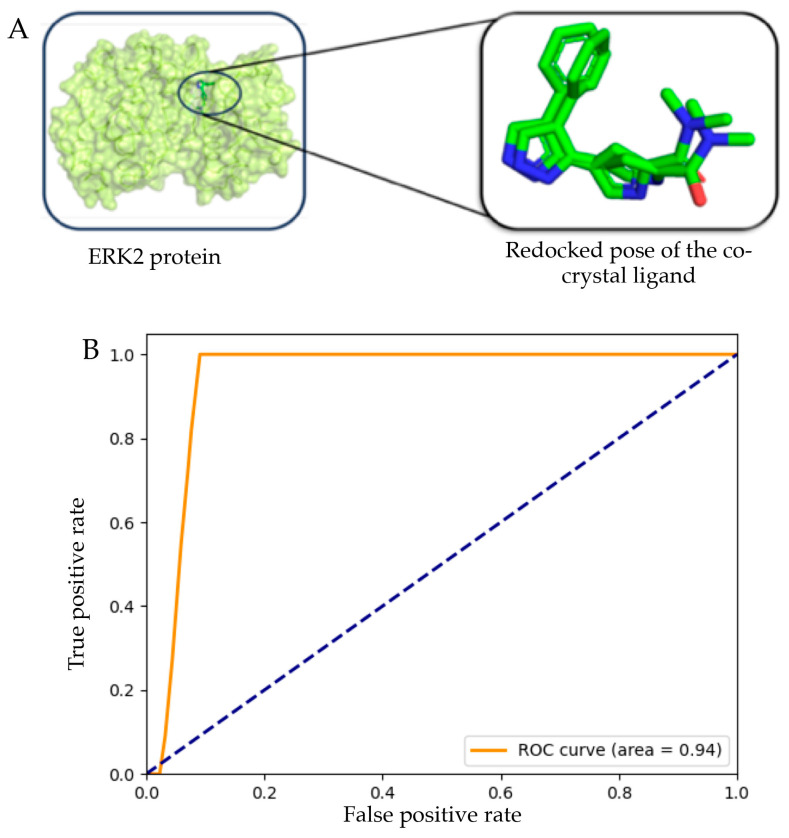
Validation of molecular orientations: (**A**) Ligand pose superimposition verifying the binding site through the re-docking approach, (**B**) ROC of the active vs. decoys fulfilling the binding affinity.

The molecules retained after the similarity search were further screened using molecular docking with AutoDock vina v1.2 (ADV) [28]. Molecular docking is a computational technique used in structural biology and drug discovery to predict and identify the optimal binding orientation of a ligand to a protein receptor, thereby forming a stable complex. Before the molecular docking, the docking protocol was validated using self-docking and decoy-set approaches to ensure that the selected docking parameters could generate conformations of new molecules comparable to the crystallographic conformation. The redocked co-crystal structure was superimposed on the original crystalized conformation, as shown in Figure 3A. Upon superimposition, the root-mean-square deviation (RMSD) between the docked pose and the co-crystal conformation was 0.87 Å, clearly validating the binding site. Further, the decoy set validation approach was considered, in which 15 active compounds were considered, and a total of 750 decoy compounds were generated from them using the DUD-E decoy generator [29]. Combined active and decoy compounds were docked using the best docking parameters found in the self-docking approach. After docking, the analysis was performed by several statistical parameters, such as accuracy, area under the curve (AUC) of the receiver operator characteristics (ROC), and specificity calculated from the confusion matrix. In particular, the accuracy was 0.825, with a ROC-AUC of 0.944, a specificity of 0.651, and a sensitivity of 1.000. The enrichment factor (EF) from the amalgamated active and decoy set yielded EF1% and EF2% values of 51.00 and 47.81, respectively. The above EF data indicate strong early enrichment of true actives in the top 1–2% of the ranked database, demonstrating that the docking protocol effectively discriminates active compounds from decoys and has reasonable predictive power for ERK2. The true-positive vs. false-positive rate was plotted and is shown in Figure 3B. The above data undoubtedly validated the docking parameters. Hence, from both self-docking and decoy set validations, it was very clear that the selected docking parameters were robust in nature, which might help to regenerate the small molecule’s conformation similar to the crystalized conformation.

A satisfactory estimation of the binding site cleared the way to perform the docking experiment for the newly derived 3000 molecules. The outcomes of molecular docking simulations are typically presented as binding energy values and the binding orientations of the compounds around the receptor’s target pocket. Here, it is to be mentioned that the co-crystal ligand on its revised docking to the protein had a binding affinity of −8.70 kcal/mol. This value was set as a screening threshold to filter the initial set of de novo compounds. This screening deemed 1818 molecules suitable for further analysis. Since these 1818 compounds were derived from the previously drawn dataset of bioactive compounds, molecular docking simulations were performed for the parent molecules to facilitate the selection of the PubChem91899270 molecule for comparative purposes. The PubChem91899270 was selected as a standard molecule based on the highest binding affinity and favorable interactions observed during the docking process. This thorough approach identifies promising candidates with the potential to effectively inhibit ERK2, laying the groundwork for further analysis and exploration of novel therapeutic avenues.

### 2.4. Pharmacokinetic, Synthetic Accessibility, and Novelty Assessment

**Figure 4:**
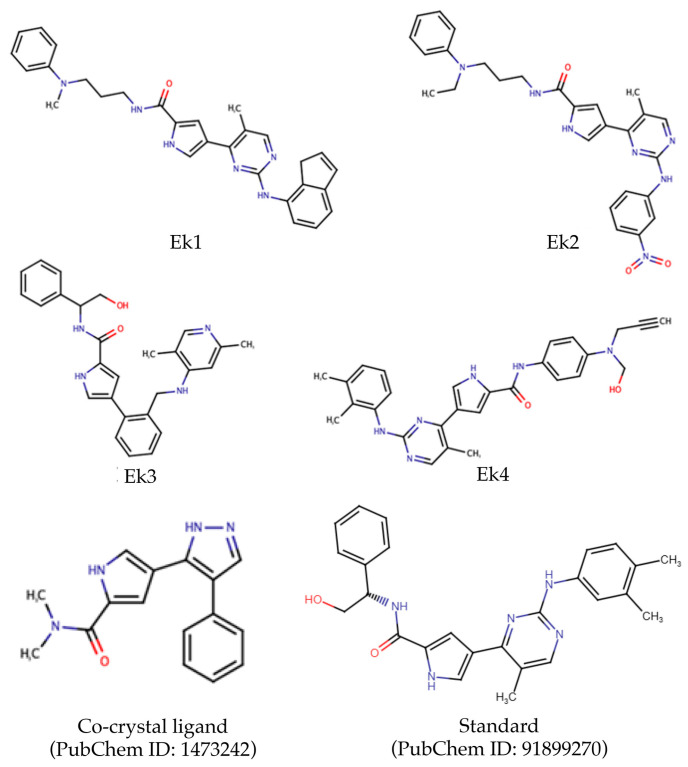
Two-dimensional (2D) chemical representation of the identified selected compounds for modulation or inhibition of ERK2 protein, co-crystal ligand, and PubChem91899270.

The ADMET study involves evaluating the pharmacokinetic properties of drug candidates in conjunction with pharmacodynamics analysis. Researchers aim to ensure that every novel drug molecule is non-toxic and safe for human use, necessitating a meticulous examination of its toxicity profile. Molecules remaining after molecular docking-based screening (a total of 1818) were considered for the ADMET analysis using the Deep-PK online web server [30], yielding corresponding ADMET data for each molecule. Specific thresholds were applied to various parameters to narrow the chemical space. Molecules exhibiting gastrointestinal absorption, skin permeability, and blood–brain barrier permeability values of ≥90, <−2.5, and <−1, respectively, were considered for further analysis. Further, CNS permeability, minnow toxicity, and maximum tolerated dose were evaluated, with desirable ranges set at <−3.0, ≥−0.30, and ≤0.477, respectively. By applying the above criteria, 26 molecules were retained for toxicity assessment. The synthetic accessibility of each of the above retained 26 compounds was assessed. The synthetic accessibility score cut-off was set at 5; i.e., molecules with a synthetic accessibility score ≤ 5 are considered moderate to synthesize. Out of 26 molecules, 4 showed ease of synthesis and were considered potential molecules for ERK2. The pharmacokinetic parameters corresponding to the SA of these selected molecules and SMILES notation are tabulated in Table 1. For convenience, these four candidate compounds, hereafter termed ‘Ek’, are prefixed with a sequential serial number in a subsequent section. The two-dimensional (2D) representation of the final selected molecules, along with the co-crystal ligand and PubChem91899270 molecules, is given in Figure 4. The novelty and uniqueness of each molecule were explored using exact-matching searches across three publicly available small-molecule databases, including PubChem [31], ZINC [32], and ChEMBL [33]. All four molecules (Ek1, Ek2, Ek3, and Ek4) were found to be unique and novel, with no matches in any of the three databases.

Further, the pharmacophore features of each molecule and PubChem91899270 were explored using the Python RDKit 2015.09.5 [24] and are given in Table S2 and Figure S1 (Supplementary data). All four proposed molecules and PubChem91899270 were found to be rich in crucial pharmacophoric features, including hydrogen bond acceptor (HBA), hydrogen bond donor (HBD), hydrophobic (HY), and ring aromatic (RA). It can be observed that Ek1, Ek2, Ek3, and Ek4 showed pharmacophoric features comparable to those of PubChem91899270, suggesting that the selected molecule possesses a significant number of pharmacophoric features that may facilitate potential binding to ERK2.

### 2.5. Binding Interactions Analysis and Absolute Binding Free Energy

Subsequent validation of the correct identification of molecules is performed using K_DEEP_. The set of screened de novo, co-crystal, and bioactive molecules was submitted to the K_DEEP_ server. Table 2 briefly tabulates the binding affinity between the two platforms. The residual interaction corresponding to the binding affinity value is also specified. In terms of both affinity and coordination with the pocket-forming residues, the newly identified candidate molecules exhibit a pattern similar to that of the PubChem91899270 and the co-crystal ligand.

**Figure 5:**
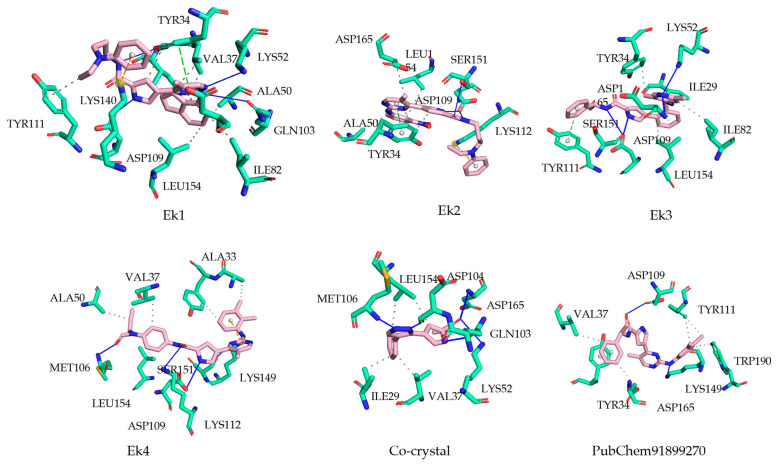
Three-dimensional (3D) interactions of molecular docking of top-selected complexes of selected compounds.

The complexes were subjected to PLIP (Protein Ligand Interaction Profiler) [34] and observed through PyMol. The intermolecular interactions observed during molecular docking are shown in Figure 5. Ek1 molecule shows hydrophobic interactions with Ile29, Val37, Tyr34, Ala50, Leu154, Ile82, Asp165, and Tyr111 and perfectly binds in the hydrophobic cavity of ERK2 protein. Compared with the co-crystal ligand molecule and the PubChem91899270 molecule, which exhibit hydrophobic interactions, Ek1 shows similar amino acid interactions involving Ile29, Val37, and Leu154. Ek2 and Ek3 molecules bind Tyr34, Ala50, Leu154, and Asp165 as hydrophobic interactions, which play a major role in inhibiting ERK2 protein. Hydrogen bond formation between the protein hydroxyl group and ligand carbonyl or hydroxyl group makes interactions stronger to bind the protein with inhibitory action, so the co-crystal ligand shows hydrogen bonds with Lys52, Gln103, Asp104, Met106, and Asp165 amino acids of the protein. In contrast, PubChem91899270 molecules interact with the Asp109 and Lys149 residues of the protein via hydrogen bonds. The Ek1 molecule forms hydrogen bonds with Tyr34, Lys52, Asp109, and Gln103, with Lys52, Gln103, and Asp109 also forming common hydrogen bonds with co-crystal ligands and PubChem91899270 molecules. Ek2, Ek3, and Ek4 molecules also interact similarly with PubChem91899270 molecules, with π–cation and π-stacking observed between mostly heteroaromatic or aromatic ligand molecules. It mostly interacts with the benzene rings of ligand molecules, forming π–π interactions. Ek1 and Ek2 molecules showed π-staking interaction with the Tyr34 amino acid of the protein, which is similar to the π-staking interaction shown by PubChem91899270 molecules. Ek2 and Ek3 show π–cation interaction with Lys112 and Tyr111 protein amino acids, respectively. Based on the above interaction profile, the proposed four molecules are capable of inhibiting and binding to the active sites of the ERK2 protein in the novel drug discovery process.

A previously reported study found that active sites of the ERK2 protein are Ile29, Tyr34, Val37, Ala50, Ile82, Tyr111, Leu154, and Asp165. The above amino acid residue is essential for inhibiting ERK2 and preventing the development of harmful diseases. After analysis of binding interactions, it was found that Val37, Lys62, Met106, and ASP165 amino acids show the most similar interactions with the ERK2 protein in the four proposed compounds compared to the co-crystal ligand and PubChem91899270 molecule.

### 2.6. Diffusion-Based Docking by DiffDock

**Figure 6:**
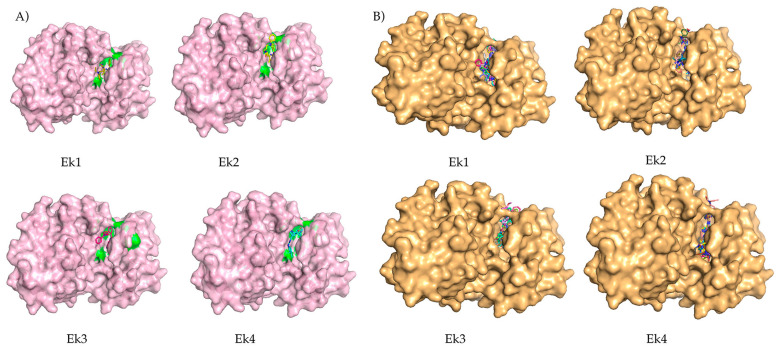
The comparison of the ligand pose in the binding pocket of the screened molecules: (**A**) Surface view of the protein as seen in PyMol, to the resulting complexes of ADV. The green colour indicates the binding pocket. (**B**) DiffDock binding poses of the same compounds based on the confidence score.

DiffDock [21] is a diffusion-based computational approach utilized in molecular docking studies to generate multiple confidence poses of a protein–ligand complex. DiffDock provides a set of confidence poses, each representing a potential ligand binding mode within the protein’s active site or binding pocket. These poses are ranked based on their calculated binding energies and other relevant parameters. In this study, DiffDock was used to investigate the binding interactions of four selected compounds with the ERK2 protein. The results of the DiffDock simulations indicated that these compounds are within the protein’s active pockets, suggesting potential inhibitory activity against ERK2. The confidence scores of the selected compounds are given in Table 3. The results obtained from this graph-based algorithm serve as a validation of the previous output from ADV. In Figure 6, we compared the poses of the ligand corresponding to the binding pocket.

### 2.7. Comparative Study of Proposed Molecules with ERK2 Molecules Under Clinical Trials

The molecules proposed in the present study were compared with ERK2 inhibitors currently in clinical trials, focusing on binding affinity and ADMET properties. LY3214996 was identified as a potent ERK1/2 inhibitor evaluated in combination with abemaciclib in a Phase 2 trial [35]. BVD-523 is a selective ERK1/2 inhibitor demonstrating promising preclinical activity, particularly against BRAF^V600E-mutant melanoma in xenograft models [16]. It is presently being assessed in clinical trials aimed at overcoming resistance to BRAF and MEK inhibitors by targeting reactivated ERK signaling pathways. MK-8353, an orally administered ERK1/2 inhibitor, has been tested in Phase I trials involving patients with advanced solid tumors [36]. The compound exhibited antitumor activity, notably in BRAFV600-mutant melanoma, with partial responses being observed in a subset of patients.

The binding energies for LY3214996, BVD-523, and MK-8353 were determined using molecular docking with ADV and DiffDock. Moreover, the absolute binding affinity was also calculated from K_DEEP_. All binding energies are presented in Table S3 (Supplementary file). Additionally, the ADMET analysis was conducted, with parameters provided in Table S3 (Supplementary file). Upon careful examination, it is evident that the binding energies obtained from ADV for Ek1, Ek2, Ek3, Ek4, LY3214996, BVD-523, and MK-8353 are −10.50, −10.20, −9.50, −9.50, −8.72, −8.91, and −9.12 kcal/mol, respectively. The aforementioned binding energy elucidates the enhanced binding affinity of the proposed molecules for ERK2, in contrast to those currently undergoing clinical trials. In a similar manner, the absolute binding affinity of the proposed molecules was determined to fall within the range of −5.23 to −6.38 kcal/mol. In contrast, the binding affinities for LY3214996, BVD-523, and MK-8353 were measured at −5.03, −5.27, and −7.90 kcal/mol, respectively. The absolute binding affinity was similar to that of the proposed molecules and the ERK2 compounds used in clinical trials. The binding energies obtained from DiffDock were recorded as −1.32, −1.49, −2.21, −1.93, −0.77, −2.13, and −1.83 kcal/mol for Ek1, Ek2, Ek3, Ek4, LY3214996, BVD-523, and MK-8353, respectively. DiffDock also demonstrated a comparable binding affinity for both sets of molecules. The ADMET analyses of the proposed molecules exhibited a pattern that closely resembles that of the ERK2 molecules in clinical trials. Therefore, it is evident that the molecules proposed in the current study exhibit superior or comparable binding affinity and ADMET parameters to those of LY3214996, BVD-523, and MK-8353.

### 2.8. Selectivity Analysis

The ATP-binding sites in kinases are highly similar, which may cause selectivity issues with the newly designed ERK2 molecules. To assess the selectivity and specificity of the newly designed final molecules, Ek1, Ek2, Ek3, and Ek4 were docked into the ERK1 protein (PDB ID: 4QTB). The similarity of the ATP-binding sites of ERK1 and ERK2 (PDB ID: 2OJG) was assessed using the ProBiS server1.0 [37], yielding a Z-score of 3.27, indicating high similarity. The binding site comparison showed that the active sites of ERK2 and ERK1 consist of almost identical core features in the hinge region of the catalytic loop. On the other hand, it displays localized differences in the solvent-exposed rim and hydrophobic pocket that could be targeted for selective inhibitor development. The superimposed ERK2 and ERK1 binding sites are given in Figure S2 (Supplementary data). Ek1, Ek2, Ek3, and Ek4 were docked in the ERK1 protein, and the binding energy was recorded. It was found that Ek1, Ek2, Ek3, and Ek4 had binding affinity towards ERK1 with binding energies of −7.40, −7.30, −7.10, and −8.10 kcal/mol, respectively. Although the binding sites of ERK2 and ERK1 were found to be highly similar, the binding affinity was seen as much higher when Ek1, Ek2, Ek3, and Ek4 bind to ERK2. The above observation suggested that the final selected molecules were more selective towards ERK2.

### 2.9. Molecular Dynamics Simulation Analysis

**Figure 7:**
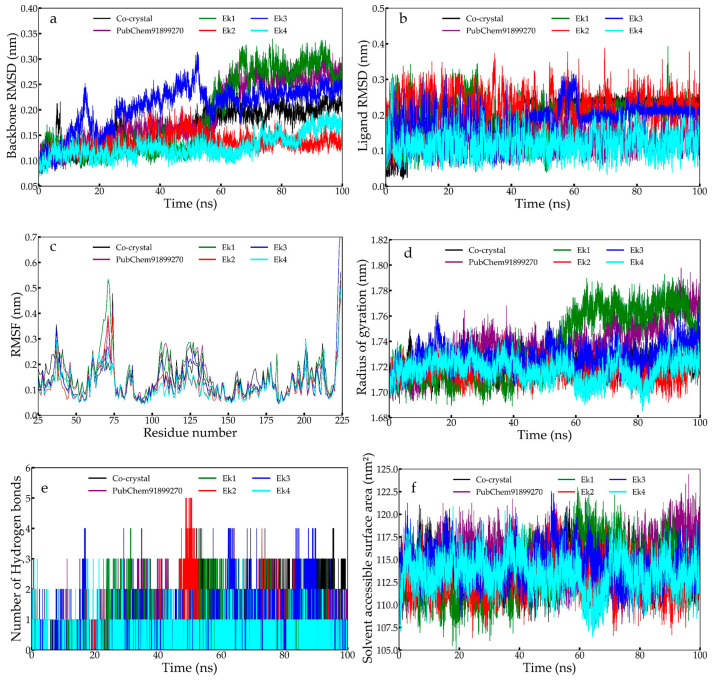
MD Simulation analysis of selected compounds, co-crystal, and PubChem91899270 molecules is shown as (**a**) Ligand RMSD, (**b**) Protein backbone RMSD, (**c**) The radius of gyration, (**d**) RMSF, (**e**) No. of hydrogen bonds, and (**f**) SASA.

After the ADMET studies, the final ligands were subjected to MD simulations for 100 ns. Gromac2023.4 was used to perform MD simulations and investigate the stability of protein–ligand complexes. Post-MDS analysis, the protein backbone RMSD (Figure 7a), ligand RMSD (Figure 7b), root-mean-square fluctuation (RMSF) (Figure 7c), radius of gyration (RoG) (Figure 7d), hydrogen bond analysis (Figure 7e), and solvent accessible surface area (SASA) (Figure 7f) were extracted from the trajectories throughout the simulation period.

#### 2.9.1. Protein Backbone RMSD

The protein backbone RMSD suggested low fluctuations for the complexes Ek4 and Ek2 throughout the simulation. In contrast, complex Ek1 initially exhibited lower fluctuations until 60 ns, then increased, reaching about 0.30 nm. Complex Ek3 remained unstable throughout the simulation period, while the PubChem91899270 and the co-crystal followed a similar pattern until 60 ns. After that, the PubChem91899270 showed greater deviation, while the co-crystal remained stable. No extreme variations in the average RMSD were observed for any candidate complexes in Table 4.

#### 2.9.2. Ligand RMSD

Similar trends were observed with the ligand RMSD; all the complexes remained pretty much stable and converged during the simulation span, while complex Ek1 fluctuated in the middle of the simulation run with higher RMSD ranging from 0.2 to 0.3 nm between 0 and 50 ns span; later it converged for the rest of the MD simulation. Complex Ek3 showed minor fluctuations between 40 and 60 ns, and the rest remained stable throughout. The co-crystal showed a steep rise in the RMSD in the later stage, reaching 0.28 nm at 40 ns. While complexes Ek4, Ek2, and the PubChem91899270 were highly stable and converged throughout the MD simulation. Therefore, it was clear that no significant deviation of the ERK2 backbone was achieved due to the incorporation of the newly designed molecules.

#### 2.9.3. Constituent Residual RMSF

The RMSF parameter is of great importance for investigating the contribution of individual amino acids to the stability of a protein-ligand complex. It measures the fluctuation of each amino acid backbone relative to its initial orientation in the native state during the simulation. The RMSF of each amino acid of ERK2 is bound with Ek1, Ek2, Ek3, Ek4, and the co-crystal, and the PubChem91899270 is given in Figure 7d. Almost all the complexes exhibit substantial fluctuations between the 50th and 75th residues during the MD simulation, with a slight increase towards the end. Other than that, not a single trajectory is found to fluctuate abnormally. All of them fluctuate below 0.5 nm, which is relatively low compared to the RMSF profile, indicating the stability of the complexes.

#### 2.9.4. Radius of Gyration

Another important MD simulation trajectory analysis parameter is RoG, which assesses the system’s compactness and rigidity in dynamic states. For better understanding, the average, maximum, and minimum RoG for each complex were calculated and given in Table 4. The difference between the maximum and minimum RoG may provide better insight into the system’s rigidity during the simulation. Except for ERK2 bound with Ek1, all other complexes showed a steady variation in RoG around 1.74 nm. RoG of ERK2 bound with Ek1 was seen to deviate a bit higher than other complexes, but no abnormal deviation was observed anywhere. The visual observation also perfectly explains the rigidity and compactness of each system.

#### 2.9.5. Intermolecular Hydrogen Bonds

In MD simulations, hydrogen bonds between the protein and the ligand are crucial for holding the ligand within the active site cavity. In most frames across all six simulated systems, at least one hydrogen bond is present (Figure 7e). A few frames show no hydrogen bonds; in those cases, the ligands are held by non-hydrogen bonds. Ek4 and Ek3 show a maximum of four hydrogen bond interactions for a certain period during simulation. In contrast, the other five hits (Ek1, Ek2, co-crystal, and PubChem91899270) exhibited consistent 2–3 hydrogen bond interactions throughout the simulation run.

#### 2.9.6. Solvent Accessible Surface Area

SASA is the area of the protein that is exposed enough to interact with the neighboring solvent molecules. A higher SASA value indicates an increase in the protein’s volume, while a low fluctuation is expected throughout the simulation. The binding of any small molecule can alter SASA and significantly impact protein structure. The SASA values of all the complexes are found to be lower in most frames than those of the co-crystal and the PubChem91899270. Most of the complexes exhibit irregular fluctuations over 40–80 ns. The average SASA values for all systems are 113.87, 113.19, 114.25, 113.48, 113.77, and 115.08 nm^2^ for Ek1, Ek2, Ek3, Ek4, co-crystal, and PubChem91899270, respectively. The first- and second-lowest average SASA values are found for the Ek2 and Ek4 complexes.

#### 2.9.7. Principal Component Analysis

**Figure 8:**
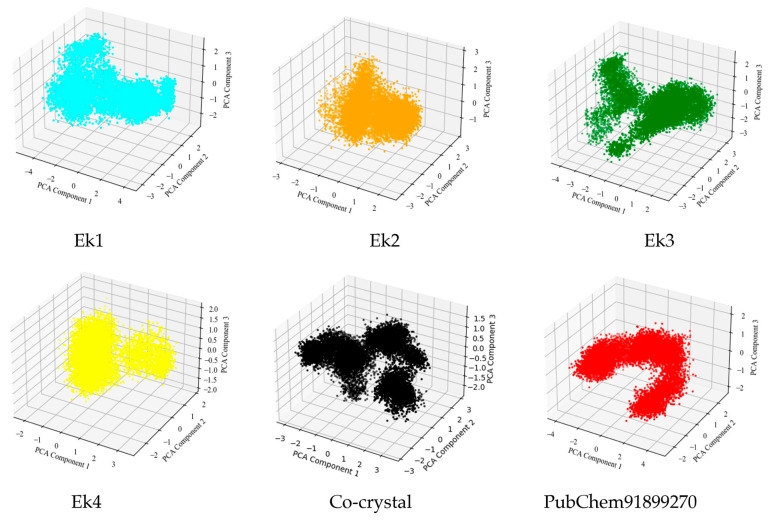
The principal component analysis of the identified hits, co-crystal ligand, and PubChem91899270 compounds of the ERK2 protein.

Principal component analysis (PCA) is used to analyze structural and energy data from MD simulations of protein–ligand complexes. PCA is often applied to molecular dynamics trajectories to extract the large-scale conformational motions of a protein. The PCA plot in Figure 8 reveals different cluster formations. Each point on the plot represents a specific conformation of the molecule captured during the simulation trajectory, projected into this three-dimensional principal component space. The distribution of points along each principal component axis reflects the extent of conformational changes captured by that component. Figure 8 Ek1 (cyan), Ek2 (orange), and the PubChem91899270 indicate tight clusters, indicating less flexibility during the simulation. The rest of the co-crystal (black), Ek3 (green), and Ek4 (yellow) form a more spread-out cluster, indicating greater flexibility.

#### 2.9.8. Free-Energy Landscape Analysis

**Figure 9:**
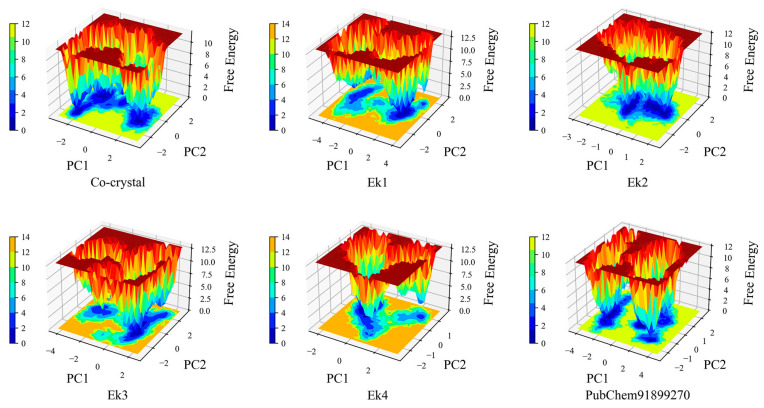
Free-energy Landscape graphs of Ek1, Ek2, Ek3, and Ek4, along with the co-crystal ligand and the PubChem91899270 molecule.

The free-energy landscape (FEL) was used to calculate and predict the thermodynamic stability of complexes. The free energy of ERK2 is evaluated using FEL analysis in Figure 9. In the free-energy landscape, the ERK2 protein undergoes more conformational changes, creating additional minima. The FEL graph shows flat minima, indicating the stability of protein–ligand complexes. Free landscape energy calculations for selected proposed compounds, PubChem91899270 molecules, and co-crystal molecules were analyzed. After assessing the Gibbs free energy of all selected compounds, flat minima were observed in four proposed compounds, similar to the flat minima of co-crystal ligands. The analysis results above indicate that the ERK2 protein is significantly more stable in the presence of ligand molecules.

#### 2.9.9. Calculation of Binding Free Energy Through the MMGBSA Approach and Per-Residue Decomposition Energy Analysis

It is a computational approach used in drug discovery to estimate the free energy of binding between molecules, such as a ligand and a protein receptor. The binding free energies of selected molecules, along with subsequent analysis of PubChem91899270 and co-crystal ligands, were calculated from MD trajectories using the MMGBSA method. The entire trajectory of each system was considered to extract frames at five intervals. The binding free energy of the proposed compounds, along with the PubChem91899270 molecule and co-crystal ligand, is depicted in Table 5.

The binding free energies of the Ek1, Ek2, Ek3, and Ek4 molecules were found to be −21.20, −3.35, −22.32, and −26.81 kcal/mol, respectively. PubChem91899270 molecule and co-crystal ligands also show binding free energy as −12.39 and −13.78 kcal/mol, respectively. Among the selected four molecules, the Ek4 molecule shows the highest binding free energy, which is higher than that of the PubChem91899270 and co-crystal ligands. Most proposed compounds have higher binding free energies than the PubChem91899270 and co-crystal ligands. So, this suggests that the final four selected molecules have excellent capacity to modulate or inhibit ERK2 activity and may be useful in ERK2-associated cancer therapeutics.

**Figure 10:**
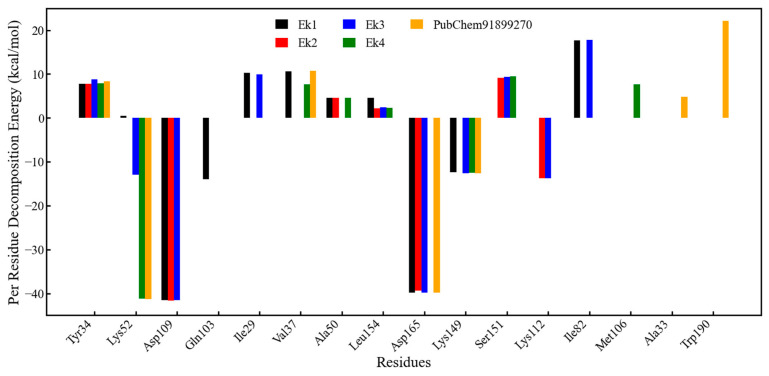
Per-residue decomposition energy of Ek1, Ek2, Ek3, Ek4, and PubChem91899270.

It was observed that Ek2 showed a much lower binding affinity of −3.35 kcal/mol, which is lower than that of PubChem91899270, as well as Ek1, Ek3, and Ek4. To explore possible reasons for the low affinity of Ek2 for ERK2, a per-residue decomposition of the binding energy for all interacting amino acids was performed and is shown in Figure 10. Ek2 was found to show affinity towards Asp109 and Asp165 with about −40 kcal/mol, whereas Lys112 showed affinity with about −10.00 kcal/mol. Except for the above three, no other amino acids showed a favorable binding affinity for ERK2. Moreover, Tyr34, Ala50, Ser151, and Leu154 showed unfavorable binding affinity for Ek2. Several other amino acids were also found to be neutral for the Ek2. A very small number of ERK2 amino acids showed favourable binding affinity for Ek2, which might be the possible reason for the lower affinity of Ek2 towards ERK2 in the MM-GBSA approach. The remaining three (Ek1, Ek3, and Ek4) molecules and PubChem91899270 showed favorable binding affinity for a significant number of amino acids in ERK2.

### 2.10. Free-Energy Perturbation

To assess the binding and unbinding energies of the molecules, the highest (Ek1) and lowest (Ek2) affinity molecules among the final four selected molecules, along with the PubChem91899270 molecule, were used for the FEP approach. The rationale behind the above selection was to determine whether the best and least-affinity molecules in the MM-GBSA study also replicate this pattern in the FEP-based binding energy. Moreover, Ek1 and Ek2 were found to exhibit higher and lower affinities, respectively, compared with PubChem91899270 in the MM-GBSA study, and these should also be retained in the FEP study.

**Figure 11:**
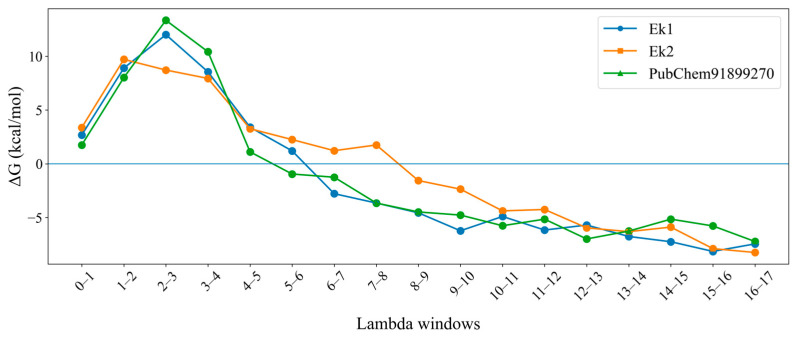
The ΔG energy differences versus 17 lambda windows graphs of Ek1, Ek2, and the PubChem91899270 molecule.

The MD simulation according to the FEP setup was carried out with 17 λ windows, and the binding energy was recorded. After successful completion and compilation, it was found that ΔG for Ek1, Ek2, and the PubChem91899270 was −26.85, −8.63, and −22.77 kJ/mol, respectively. The above data indicate that although Ek2 showed a lower affinity than the PubChem91899270, Ek1 showed much better affinity for ERK2 using the FEP approach. The ΔG energy difference was plotted for each λ window and is shown in Figure 11. It can be seen that, from λ windows 5–6 for both Ek1 and PubChem91899270, and 8–9 for Ek2, the ΔG tended to a negative value and never returned to a positive value. The above observation indicates that the λ windows were critical for binding stabilization.

**Figure 12:**
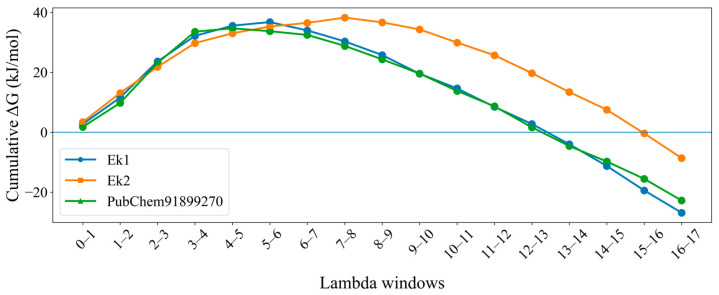
The graphic depicts the cumulative ΔG energy of Ek1, Ek2, and the PubChem91899270 molecule.

A cumulative ΔG trend line for Ek1, Ek2, and PubChem91899270 molecules was plotted and is shown in Figure 12. In the case of Ek1, the cumulative curve was smoother than that of Ek2 and PubChem91899270, suggesting that Ek1 was the most favorable binder. Hence, FEP studies confirm that all three molecules showed strong affinity; Ek1 was the best binder towards ERK2.

## 3. Discussion

In this study, a de novo approach was used, employing the deep learning-based DeLA-Drug tool to generate new inhibitors targeting the ERK2 protein, a key component in cancer-related signaling pathways. Starting from 78 known active compounds with strong binding affinity for ERK2, we created over 10,000 new molecules and filtered them using fingerprint-based similarity (Tanimoto coefficient ≥ 0.6), keeping 3000 structurally related candidates. Rigorous molecular docking with ADV, validated by self-docking (RMSD 0.87 Å) and decoy-set enrichment (ROC-AUC 0.944), identified 1818 molecules with better binding energy than the reference co-crystal ligand (−8.70 kcal/mol). Follow-up ADMET filtering using Deep-PK and synthetic accessibility assessment (SA ≤ 5) narrowed the list to four promising candidates (Ek1–Ek4), which showed favorable pharmacokinetic profiles, low predicted toxicity, and reasonable ease of synthesis. Interaction analysis showed that these molecules bind the ERK2 ATP-binding pocket through key hydrophobic contacts (e.g., Val37, Leu154, Asp165) and hydrogen bonds (e.g., Lys52, Gln103, Asp109), similar to patterns observed with the co-crystal ligand and the PubChem91899270 reference compound. Additional validation with K_DEEP_ and diffusion-based DiffDock docking confirmed consistent binding poses and comparable or better affinity scores. Remarkably, the proposed compounds exhibited stronger binding energies in ADV (ranging from −9.50 to −10.50 kcal/mol) than the clinically trialed ERK1/2 inhibitors (LY3214996, BVD-523, MK-8353), while showing similar ADMET properties and absolute binding affinities. MD simulations over 100 ns demonstrated the stability of the protein–ligand complexes. Ek2 and Ek4 showed particularly low fluctuations in protein backbone RMSD, ligand RMSD, RoG, and RMSF profiles, with consistent hydrogen bonding (2–4 bonds) and lower SASA values, indicating a compact, well-buried binding mode. MMGBSA calculations produced favorable binding free energies, with Ek4 reaching −26.81 kcal/mol, significantly better than the PubChem91899270 (−12.39 kcal/mol) and co-crystal ligand (−13.78 kcal/mol). FEP further confirmed Ek1 as the strongest binder with an absolute value of ΔG = −26.85 kJ/mol, outperforming the PubChem91899270. Collectively, these multi-level computational results indicate that the four de novo designed molecules exhibit enhanced binding affinity, structural complementarity, dynamic stability, and drug-like properties relative to existing ERK2 inhibitors. These findings position Ek1–Ek4 as compelling candidates for further experimental validation as potential therapeutics in ERK2-driven cancers.

### Limitations

The present study was limited by the inherent approximations of the CHARMM36 force field and the SwissParam-derived parameters, as well as the relatively short 100 ns MD timescale, which may not fully capture slower conformational transitions or long-range dynamic effects. Additionally, MM/GBSA binding free-energy calculations ignore explicit entropic contributions. The limitations mentioned above were addressed to some extent through orthogonal validation methods, such as absolute FEP. Implementing metadynamics could provide more robust, entropy-aware estimates of binding affinity. Moreover, the computational methods, including high-throughput virtual screening and de novo designing of small molecule inhibitors, can be powerful tools for drug discovery; it is important to recognize their limitations. In silico predictions, while informative, are often less time-consuming and cost-effective and require experimental validation to confirm their accuracy and potential therapeutic value. Rigorous experimental validation, including biochemical and cellular assays, is essential to confirm the potency, selectivity, and efficacy of the identified inhibitors. In vitro studies can help assess the inhibitors’ ability to inhibit ERK2 activity, their impact on downstream signaling pathways, and their potential off-target effects. Comprehensive preclinical studies, including in vivo animal models, are also useful and necessary to properly evaluate the safety, pharmacokinetics, and pharmacodynamics of the identified lead compounds. These studies will help identify potential toxicities, optimize dosing regimens, and assess the therapeutic efficacy of the identified inhibitors in relevant disease models, including cancer. Moreover, collaborating with pharmaceutical companies to gain better insight into bioavailability data can provide access to advanced drug discovery and development expertise at a very early stage, as well as the necessary resources to proceed to clinical trials. Precisely, pharmaceutical industry partners can help with better formulation development, manufacturing, and regulatory affairs, accelerating the translation of identified promising compounds into clinical candidates. By addressing these key considerations and fostering strong collaborations, it is possible to bridge the gap between computational predictions and clinical reality, ultimately overcoming limitations and advancing the development of novel and effective ERK2 inhibitors for therapeutic applications.

## 4. Materials and Methods

### 4.1. Selection and Structural Evaluation of the ERK2 Protein

The X-ray crystallographic structure of the ERK2 protein was collected from the PDB. Among the available crystal structures in the PDB, several criteria were considered, including source organism, experimental method, resolution, the difference between the observed and calculated R-values, presence of a mutation, and the bound co-crystal ligand, to consider the correct structure. In particular, structures with high resolution, small differences between observed and calculated R-values (i.e., considerably less than or equal to 0.05), and no mutations unless they are essential for biological significance, might be suitable for any in silico study. Among several entries, PDB ID: 2OJG [27] was identified as *Homo sapiens*, with a resolution of 2.00 Å, a difference of 0.004 between the observed and calculated R-values, no mutations, and a small molecule bound at the active site. Thus, PDB ID: 2OJG [27] was selected for the current study. The selected protein structure consists of 380 amino acid residues with a co-crystal ligand, N, N-dimethyl-4-(4-phenyl-1H-pyrazol-3-yl)-1H-pyrrole-2-carboxamide (deposited in the repository as 19A). 19A can be identified in the PubChem database under ID 1473242 and CAS ID 321553-20-2. The molecule consists of a pyrrole ring linked to a phenyl-substituted pyrazole ring, used to study enzyme interactions, such as MAP kinase (ERK2) inhibition, and explored for potential applications in medicinal chemistry and materials science due to its heterocyclic structure [28]. A follow-up literature survey indicated that the co-crystal ligand is bound to the pocket formed by Ile29, Val37, Lys52, Gln103, Asp104, Met106, Leu154, and Asp165, which is the ATP-binding site of ERK2 [16]. The re-evaluation of the binding site was confirmed by self-docking of the co-crystal ligand and the protein, thereby rendering it the active site.

### 4.2. Reported Existing ERK2 Inhibitors Collection and De Novo-Based Compound Library Creation

Known active compounds for a specific target serve as valuable blueprints in drug discovery, offering crucial insights into the molecular features necessary for biological activity. By thoroughly analyzing their structures, pharmacophores, and structure–activity relationships (SAR), researchers can identify key functional groups, binding motifs, and privileged scaffolds that facilitate target engagement. These insights allow for the rational modification of existing leads to create structurally novel derivatives with improved potency, selectivity, pharmacokinetic properties, and reduced toxicity. By using the key features of existing active inhibitors and designing new potential chemical entities, a set of 78 active ERK2 inhibitors with biological activity (K_i_) spanning 0.4 to 5 nM was collected from the literature. DeLA-Drug captures the syntax of SMILES strings for compounds in the input dataset and introduces a new strategy, sampling with substitutions (SWS), that generates molecules from a single user-defined query compound. The method produced drug-like analogues of known active compounds using a training procedure based on the bioactive molecules. DeLA-Drug [11] enables rapid generation of focused libraries for high-throughput screening without the time-consuming fine-tuning required by other methods. The 78 active compounds were given as input for generating drug-like analogues (de novo) compounds. The DeLA-Drug-generated compounds were used for similarity search-based analysis, as explained hereafter.

### 4.3. Precision of the De Novo Library Compounds by Fingerprint-Based Similarity Search

The Similar Property Principle (SPP) refers to the phenomenon in which molecules that share structural similarities exhibit comparable properties [38]. This principle is a core concept in drug discovery, stating that small structural modifications to an active compound are likely to maintain its biological activity against a specific target [30]. In this context, a similarity search was conducted utilizing molecular fingerprints derived from a molecule’s chemical attributes, including its atom types, bonding patterns, and bonded interactions. In particular, the Python RDKit [24] tool was used for the similarity search. The DeLA-Drug-generated de novo library was scanned for similarity with the active ERK2 inhibitors. Subsequently, the molecules had a Tanimoto similarity coefficient of 0.6 or higher and were retained for further investigation.

### 4.4. Molecular Docking of De Novo-Generated Library, Co-Crystal Ligand, and Standard Compounds

Utilizing computational methodologies for the virtual screening of compound libraries has become prevalent in modern drug discovery processes. When a suitable target structure is available, molecular docking is a valuable tool for distinguishing potential binders from non-binders across extensive chemical space. This approach significantly reduces the number of compounds requiring experimental validation. The process of docking small-molecule compounds into the receptor’s binding site and assessing the binding affinity of the resulting complex is a critical aspect of structure-based drug design methodologies [39]. In the current study, ADV [28] was used to initiate the molecular docking analysis. The docking procedure was iterated for three sets of compounds against the same receptor. The three sets are (a) the active inhibitors, (b) the de novo dataset, and (c) a set of decoys corresponding to the active inhibitors, set up as a validation criterion of the interaction. For the numerous docking candidate lists, the procedure was carried out in parallel using the Message Passing Interface (MPI-Vina) [33]. Each time, the set of docking molecules was provided with a configuration file that specifies their storage path and the grid parameters for allocating the interaction space. The grid coordinate was set to −13.299, 9.372, and 40.194 Å, with dimensions of 60 × 64 × 58 Å^3^ along the X, Y, and Z axes, respectively.

#### 4.4.1. Preparation of Protein and Small Molecules

The selected crystal structure of ERK2 was methodically checked and prepared for molecular docking using the AutoDock tools (ADT) [40]. The unwanted components, including the co-crystal water and small molecules, ions, etc., were removed. The missing atoms were identified and added. The hydrogen atoms and Gateigier charges were added. Finally, the protein was saved in .pdbqt format after assigning the AD4 (Autodock4) atom types. The de novo generated molecules and redrawn co-crystal ligand were prepared using OpenBabel [41], a freely available tool installed on the Linux platform. Each molecule was considered, and hydrogen and Gasteiger charges were added. The 3D coordinates were generated, and the molecules were saved in .pdbqt format for molecular docking using ADV.

#### 4.4.2. Molecular Docking Protocol Validation

Validation of the docking protocol prior to using it for any new molecule is an essential process for assessing the acceptability of docking outcomes. Self-docking and decoy set validation methods are widely accepted and used in molecular docking studies. In the self-docking approach, the co-crystal-bound ligand (19A) was redrawn and docked at the same site where it was bound. The best pose was extracted and superimposed on the co-crystal conformation of 19A. It is reported that the docking protocol, which generates a superimposed root-mean-square deviation (RMSD) of ≤2 Å between the co-crystal conformation and the best docked pose, can generate a pose similar to the crystal structure. In decoy set validation, a set of active and inactive molecules was generated for the ERK2. The inactive molecules (decoys) were collected from the generated using DUD-E, a decoy web tool [42]. A set of 15 active compounds and 750 decoys was combined for the docking study. The objective of the decoy set was to determine whether the considered protocol can identify active molecules with higher binding affinity than inactive molecules. The protocol, developed using the self-docking approach, was re-validated using decoy set validation. After docking the amalgamated sets, accuracy and AUC were calculated.

### 4.5. Absolute Binding Free-Energy Estimation Using K_DEEP_

The absolute binding affinity of each molecule was determined using the machine-learning (ML)-assisted K_DEEP_ tool [43]. This tool predicts protein–ligand affinity using 3D convolutional neural networks (3D-Deep Convolutional Neural Networks). The model is trained using a substantial dataset of protein–ligand complexes with known binding affinities. This enables the neural network to learn and comprehend the intricate relationships between these structures and their corresponding binding affinities. Following training, K_DEEP_ can predict the binding affinity of new protein–ligand complex inputs fed to the neural network, estimating the absolute binding affinity between the protein and ligand. The prerequisites for this web-based server are the coordinated files of the ligands and the receptor molecule, and an index file listing the source path of the system. The protein can be submitted in “pdb” format, while the ligands should be submitted in “sdf” format. All required files are kept in the same location, and the compressed directory is provided to the server. Here, we have tested the de novo compounds and the active molecules to estimate the binding free energies of the complexes they form.

### 4.6. ADME and Toxicity Study

Pharmacokinetics is an important parameter to consider in the safety profile of compounds being assessed in the drug discovery process. All compounds retained from previous steps were subjected to in-depth pharmacokinetic analysis using ‘Deep-PK’, a freely accessible ML-guided tool [30] widely used for in silico ADMET prediction. In Deep-PK, graph-based signatures and a graph neural network are used. These representatives of all small molecules are submitted to the Deep-PK tools to generate different pharmacokinetic properties. Notably, important ADMET parameters were calculated for each compound, including intestinal absorption, skin permeability, BBB permeability, and CNS permeability. Along with ADME profile estimation, a toxicity study was also considered for the filtration analysis of potentially less toxic or non-toxic compounds using the same Deep-PK tool. Precisely, in the toxicity study, AMES toxicity, Minnow toxicity, Maximum tolerated dose [human], hepatotoxicity, and skin sensitization were analyzed and compared. Molecules with satisfactory pharmacokinetic and toxicity profiles were considered for further analysis.

### 4.7. Synthetic Accessibility Evaluation

An approach for assessing the synthetic accessibility (SA), or ease of synthesis, of drug-like molecules is essential across various stages of the drug discovery process. Evaluating the SA of a potential lead candidate is crucial during lead discovery, regardless of the method used to identify it. For de novo-designed molecules, experimental validation of their activity requires compound synthesis. The more difficult the synthesis of the lead candidate is, the more time and resources are needed to explore this particular area of chemical space. In that context, the SwissADME web tool [44] was used, which provides free access to a pool of fast yet robust predictive models for physicochemical properties, pharmacokinetics, drug-likeness, and the synthetic accessibility of compounds. In a web browser, the input is provided as SMILES for the compounds whose SA is to be investigated. The compounds that met the Deep-PK criteria were subjected to SwissADME for SA assessment.

### 4.8. Diffusion-Based Docking Validation Using DiffDock

DiffDock is a computational technique used in molecular docking to generate multiple high-confidence poses of a protein–ligand complex. This methodology is valuable for investigating potential binding orientations and interactions between a specific ligand and its target protein. In DiffDock, molecular docking was performed using algorithms designed to predict the most energetically favorable configurations of the protein–ligand complex. DiffDock provides a set of confidence poses, each representing a potential ligand binding mode within the protein’s active site or binding pocket. These poses were ranked based on their calculated binding energies and other relevant parameters. To perform DiffDock, input files containing the protein’s three-dimensional structure (PDB format) and the ligand (SMILES format) are required. These files were then processed using DiffDock, which runs docking simulations and generates confidence poses for the protein–ligand complex.

### 4.9. Molecular Dynamics Simulation Study and Binding Free Energy by MMGBSA

The MD simulations were conducted for the final selected ERK2 potential, along with the standard and the co-crystal. MD simulation is profoundly considered a crucial method for comprehending the dynamic nature of the protein–ligand complex. For each complex, a 100 ns MD simulation production run was performed. The MD simulation was conducted using Gromacs2023.4 [45] in a Linux operating system environment embedded with an NVIDIA^®^ GeForce RTX^TM^ 2070 and a 10th-generation Intel Core i9-10885H processor. In particular, before the simulation production was initiated, a bio-macromolecular system was prepared. Therefore, all ligand topologies were parameterized using the SwissParam tool [46], and the protein topology was generated using the CHARMM36 force field [47]. A time step of 2 fs, a constant pressure of 1 atm, and a constant temperature of 300 K were used throughout the simulation. Every protein–ligand complex was submerged in a cubic box, with a minimum distance of 10 Å between the center and the box edge, and the TIP3P water model was used to simulate the entire system. Moreover, the entire system was neutralized by adjusting the required amounts of Na^+^ and Cl^−^ ions. The steepest-descent algorithm was used to minimize all systems to address the overlap and close connections between the atoms. Before the MD simulation production phases, each system was equilibrated in NVT, followed by NPT for 5 ns. Finally, a 100 ns MD trajectory was run, and parameters such as RMSD of the protein and ligand, RMSF, RoG, inter-molecular hydrogen bonds, and SASA were calculated using MD simulation. A FEL map was generated, representing the system’s Gibbs free energy as a function of its conformations or reaction coordinates. It reveals stable states, transition barriers, and folding or binding pathways in biomolecules. The mobility of the system at the atomic level was also explored using PCA. Furthermore, the MM-GBSA approach [48] was used to calculate binding free energy from MD simulation trajectories using the gmx_MMPBSA package [49]. A total of 2000 frames from the beginning to the end of the simulation, with an interval of 5, were considered to estimate the binding free energy of selected proposed compounds and standard compounds of the ERK2 protein.

### 4.10. Free-Energy Perturbation Study

The FEP is a rigorous, physics-based computational method derived from statistical mechanics that allows precise calculation of free-energy differences between two closely related thermodynamic states [50]. It is most commonly used in drug discovery to predict the relative binding affinities of ligands. By performing alchemical transformations that gradually convert one ligand into another or into a hypothetical decoupled state through a series of intermediate λ-windows, FEP calculates the free-energy change using the Zwanzig equation, usually through MD simulations. Over the past decade, improvements in force fields, enhanced sampling techniques, and GPU-accelerated computing have made FEP one of the most accurate tools for prospective lead optimization, often achieving high accuracy and significantly speeding up rational molecular design in pharmaceutical research [51]. FEP execution includes system energy minimization for up to 5000 steps, NVT and NPT equilibration for 0.002 steps, and MD production for a 1 ns simulation. FEP was conducted with 17 lambda windows (0–16). After completing the FEP analysis, the free-energy differences (ΔG) for all 17 windows were retrieved, yielding a final ΔG value in kJ/mol. Graphs were produced for ΔG energy differences vs. lambda windows.

## 5. Conclusions

ERK2, a key serine/threonine kinase in the MAPK/ERK cascade, critically governs cell proliferation. Previously, FR180204 and SCH772984 have shown expected results in treating inflammation and cancer, respectively [17,52,53]. However, limitations associated with existing inhibitors, such as off-target effects and resistance development, necessitate exploring novel therapeutic avenues for this protein, including ERK2. In this effort, a de novo drug design method and an AI/ML-driven virtual screening technique were used to find potent and selective ERK2 inhibitors. The present study initially explores the target protein’s active site and then uses DeLA-Drug to mine new molecules that could be developed into drugs. RDKit utilization streamlined the vast chemical space into a smaller, more manageable, and more precise set. The molecular docking algorithm was tested several times. Initially, it helped to observe the binding site of the active molecules. All molecules exhibit strong binding interactions at the active sites of the ERK2 protein, including Ile29, Tyr34, Val37, Ala50, Ile82, Tyr111, Leu154, and Asp165, as compared with selected PubChem91899270 and co-crystal ligands of the ERK2 protein. Decoys were generated using DUD-E, whose interaction conformed to the binding site. The rigorous molecular interaction analyses in K_DEEP_ and DiffDock validated ADV’s results. In the cellular mechanism of ERK2 protein, Val37, Leu154, and Asp165 amino acids play a pivotal role. The selected four compounds exhibited hydrophobic interactions with these residues and bound perfectly to the hydrophobic cavity of the ERK2 protein. The binding affinity of one of the selected molecules was better than that of the PubChem91899270 molecule in FEP analyses. The complex formed by these lead compounds also shows stability proportional to that of the reference compounds. The synthetic accessibility of these leads also paves the way for in vitro potential. Hence, these lead compounds can be further evaluated in vitro to assess their therapeutic potential as new ERK2 inhibitors.

## Figures and Tables

**Table 1:** ADME Properties and synthetic accessibility of selected proposed compounds.

Compounds	SMILES	Molecular Weight	Gastrointestinal Permeability	BBB Permeability	Skin Permeability	Synthetic Accessibility
Ek1	CCN(c1ccccc1)CCCNC(=O)c1[nH]cc(c1)c1nc(ncc1C)Nc1cccc2c1CC=C2	492.62	92.37	−1.06	−2.74	3.9
Ek2	CCN(c1ccccc1)CCCNC(=O)c1[nH]cc(c1)c1nc(ncc1C)Nc1cccc(c1)N(=O)=O	499.57	91.83	−1.30	−2.73	3.6
Ek3	OC[C@@H](c1ccccc1)NC(=O)c1[nH]cc(c1)c1ccccc1CNc1cc(C)ncc1C	440.54	93.13	−0.93	−2.73	3.8
Ek4	C#CCN(c1ccc(cc1)NC(=O)c1[nH]cc(c1)c1nc(ncc1C)Nc1cccc(c1C)C)CO	480.57	90.91	−1.22	−2.73	3.8

**Table 2:** Interactions Profile of ADV and K_DEEP_ of selected compounds.

Compounds	PubChem ID	ADV (kcal/mol)	*K*_DEEP_ (kcal/mol)	Interacting Amino Acid Residues	
				Hydrogen Bond	Other Types of Interactions
Ek1	-	−10.50	−6.14	Tyr34, Lys52, Asp109, Gln103	Ile29, Val37, Tyr34, Ala50, Leu154, Ile82, Asp165, Tyr111, (Hydrophobic)/Tyr34 (π-stacking) /Lys149 (π-cation)
Ek2	-	−10.20	−5.67	Asp109, Ser151	Tyr34, Ala50, Leu154, Asp165 (Hydrophobic)/Tyr34 (π-stacking)/Lys112 (π-cation)
Ek3	-	−9.50	−5.23	Lys52, Asp109, Ser151, Asp165	Ile29, Tyr34, Ile82, Leu154 (Hydrophobic)/Tyr111(π-stacking)
Ek4	-	−9.50	−6.38	Met106, Asp109, Lys112, Lys149, Ser151	Ala33, Tyr34, Val37, Ala50, Leu154 (Hydrophobic)/Lys149 (π-cation)
Co-crystal ligand	1473242	−8.70	−8.40	Lys52, Gln103, Asp104, Met106, Asp165	Ile29, Val37, Leu154 (Hydrophobic)
Standard molecule	91899270	−10.10	−9.15	Asp109, Lys149	Val37, Tyr111, Asp165, Trp190 (Hydrophobic)/Tyr34 (π-stacking)

**Table 3:** DiffDock confidence score of the selected three compounds.

Compound Name	DiffDock Confidence Score
Ek1	−1.32
Ek2	−1.49
Ek3	−2.21
Ek4	−1.93

**Table 4:** Statistical parameters obtained from MD simulation trajectories.

Parameters		Ek1	Ek2	Ek3	Ek4	Co-Crystal	PubChem91899270
Backbone RMSD (nm)	Average	0.19	0.14	0.21	0.13	0.17	0.19
	Maximum	0.34	0.21	0.31	0.20	0.25	0.32
	Minimum	0.00	0.00	0.00	0.00	0.00	0.00
Ligand RMSD (nm)	Average	0.21	0.23	0.18	0.12	0.19	0.14
	Maximum	0.39	0.39	0.32	0.30	0.27	0.26
	Minimum	0.00	0.00	0.00	0.00	0.00	0.00
RMSF (nm)	Average	0.16	0.12	0.13	0.12	0.14	0.15
	Maximum	0.53	0.46	0.73	0.50	0.56	0.52
	Minimum	0.05	0.05	0.05	0.04	0.05	0.06
RoG (nm)	Average	1.74	1.72	1.73	1.72	1.72	1.74
	Maximum	1.79	1.75	1.76	1.75	1.76	1.80
	Minimum	1.69	1.69	1.70	1.69	1.69	1.69
SASA (nm)^2^	Average	113.88	113.20	114.26	113.48	113.78	115.08
	Maximum	123.01	121.15	122.56	120.85	121.45	124.40
	Minimum	104.87	106.35	107.87	106.43	108.12	107.85

**Table 5:** Average binding free energy and standard deviation of selected molecules with PubChem91899270 and co-crystal ligand.

Compounds	Average Binding Free Energy (kcal/mol)	Standard Deviation (±)
Ek1	−21.20	4.00
Ek2	−3.35	5.70
Ek3	−22.32	5.72
Ek4	−26.81	9.16
PubChem91899270	−12.39	9.14
Co-crystal	−13.78	4.74

## Data Availability

The original contributions presented in this study are included in the article/Supplementary Materials. Further inquiries can be directed to the corresponding author.

## Supplementary Materials

The following supporting information can be downloaded at: https://www.mdpi.com/article/10.3390/ph19020337/s1. Table S1. SMILES, PUBCHEM IDs, Ki values and binding affinity of active molecules; Table S2. Pharmacophoric features of Ek1, Ek2, Ek3, Ek3, and PubChem91899270; Table S3. Binding affinity and ADMET parameters of ERK2 inhibitors; Figure S1. Pharmacophoric features of Ek1, Ek2, Ek3, Ek3, and PubChem91899270; Figure S2. Superimposition of ERK2 and ERK1 active sites obtained from the ProBis server.

## Abbreviations

The following abbreviations are used in this manuscript:

ERK2	Extracellular-Signal-Regulated Kinase 2
MEK	Mitogen-activated protein kinase
ATP	Adenosine triphosphate
DeLA-Drug	Deep Learning Algorithm for Automated Design of Drug-like Analogues
MD	Molecular dynamics
MM-GBSA	Molecular Mechanics/Generalized Born Surface Area
FEP	Free-energy perturbation
SPP	Similar property principle
ADT	AutoDock tools
ADV	Autodock vina
AD4	Autodock4
AUC	Area under the curve
ADMET	Adsorption, distribution, metabolism, excretion, and toxicity
SA	Synthetic accessibility
RMSF	Root-mean-square fluctuation
RMSD	Root-mean-square deviation
RoG	Radius of gyration
SASA	Solvent accessible surface area
FEL	Free-energy landscape
