# Supplementary material for: Identification of Novel Extracellular-Signal-Regulated Kinase 2 Inhibitors Through Machine Learning-Driven De Novo Design, Molecular Docking, and Free-Energy Perturbation
Source: Pharmaceuticals (Basel). 2026 Feb 20;19(2):337. doi: 10.3390/ph19020337 (PMC12943845; doi:10.3390/ph19020337)
Supplement: Supplementary file 1 [file pharmaceuticals-19-00337-s001.zip › pharmaceuticals-4149158-supplementary.pdf]

Identification of Novel Extracellular Signal-Regulated Kinase 2 Inhibitors through Machine Learning-driven de-novo Design, Molecular Docking, and Free Energy Perturbation

Supplementary data

Table S1. SMILES, PUBCHEM IDs, Ki values and binding affinity of active molecules

Mol.	SMILES	PubChem ID	Ki kcal/mol	Binding affinity (kcal/mol)
A01	c1(cc([nH]c1)C(=O)N[C@H](c1ccccc1)CO)c1c(C)cnc(n1)Nc1cc(ccc1)C(=O)O	91899300	0.4	-9.1
A02	Cc1c(nc(NNNC(=O)c2c(cccc2)O)nc1)c1cc([nH]c1)C(=O)N[C@H](c1ccccc1)CO	91899297	0.5	-8.8
A03	c1(cc([nH]c1)C(=O)N[C@H](c1ccccc1)CO)c1nc(Nc2c(c(cc(c2)C)OC)C)ncc1C	60090596	1.0	-9.2
A04	c1([nH]cc(c1)c1c(C)cnc(Nc2c(c(ccc2)OC)OC)n1)C(=O)N[C@@H](c1cc(c(cc1)OC)OC)CO	70701683	1.0	-8.7
A05	c1(cc([nH]c1)C(=O)N[C@H](c1ccccc1)CO)c1c(C)cnc(n1)Nc1ccc(cc1)C(=O)O	91899305	1.0	-9.1
A06	c1(cc([nH]c1)C(=O)N[C@H](c1c(C)cc(cc1)O)CO)c1nc(Nc2c(C)cc(cc2)N)ncc1C	44481495	1.0	-9.9
A07	c1(cc([nH]c1)C(=O)N[C@H](c1c(cc(cc1)O)O)CO)c1c(C)cnc(n1)Nc1c(C)c(C)ccc1	44481493	1.0	-9.3
A08	c1(cc([nH]c1)C(=O)N[C@H](c1c(cccc1)C(=O)O)CO)c1c(C)cnc(n1)Nc1c(C)c(C)ccc1	70701746	1.0	-9.1
A09	Cc1c(nc(Nc2cc(c(c(c2)O)O)O)nc1)c1cc([nH]c1)C(=O)N[C@H](c1ccccc1)CO	91899338	1.0	-9

A10	c1(cc([nH]c1)C(=O)N[C@H](c1ccccc1)CO)c1nc(NCc2c(cc(cc2)OC)C)ncc1C	91899346	1.0	-8.9
A11	c1([nH]cc(c1)c1c(C)cnc(Nc2c3CC=Cc3ccc2)n1)SCCC(=O)N[C@@H](c1ccccc1)CC	91899266	2.0	-9.8
A12	N(C(=O)c1[nH]cc(c1)c1c(C)cnc(NCC)n1)[C@@H](c1ccc(cc1)CCCC)CO	70701672	2.0	-9.3
A13	c1(cc([nH]c1)C(=O)N[C@H](c1ccccc1)CO)c1c(C)cnc(n1)Nc1[nH]c(cn1)C#N	91899270	2.0	-10.1
A14	c1([nH]cc(c1)c1c(C)cnc(Nc2c(C)c(C)ccc2)n1)C(=O)N[C@@H](NCc1cc(ccc1)OC)CO	70701679	2.0	-8.5
A15	c1([nH]cc(c1)c1c(C)cnc(Nc2c3CC=Cc3ccc2)n1)C(=O)NCCCN(c1ccccc1)CC	91899293	2.0	-9.6
A16	c1(cc([nH]c1)C(=O)N[C@H](c1ccccc1)CO)c1c(cccc1)CNc1nc(C)ncc1C	60147038	2.0	-8.2
A17	c1([nH]cc(c1)c1c(C)cnc(Nc2cc(ccc2)N(=O)=O)n1)C(=O)NCCCN(c1ccccc1)CC	91899299	2.0	-9.3
A18	c1(cc([nH]c1)C(=O)Nc1c(cccc1)S(=O)(=O)CO)c1c(C)cnc(n1)Nc1ccccc1	91899306	2.0	-8.9
A19	N(c1nc(c(C)cn1)c1cc([nH]c1)C(=O)N[C@H](c1ccccc1)CO)n1cnc2c(NCC)ncnc12	91899312	2.0	-9.2
A20	c1([nH]cc(c1)c1c(C)cnc(Nc2ccccc2)n1)C(=O)N[C@@H](Nc1c(cc(C)cc1)C#N)CO	70701743	2.0	-9.4
A21	c1(cc([nH]c1)C(=O)N[C@H](c1ccccc1)CO)[C@@H]1N=C(N=C[C@H]1C)[C@@H]1[C@@](CC1)(c1ccccc1)OC	91899324	2.0	-8.7
A22	c1(cc([nH]c1)C(=O)N[C@H](c1ccccc1)CO)c1c(C)cnc(n1)NC(=O)Cc1[nH]cnc1	91899337	2.0	-9.8
A23	c1(cc([nH]c1)NS(=O)(=O)[C@@H](C)c1cc(C)ccc1)c1nc(Nc2ccc(C)cc2)ncc1C	70701803	2.0	-8.7
A24	c1([nH]cc(c1)c1c(C)cnc(Nc2cc(C)c(C)cc2)n1)C(=O)NCCCN(c1ccccc1)CC	91899353	2.0	-9.5

A25	c1([nH]cc(c1)c1c(C)cnc(Nc2ccc(C)cc2)n1)C(=O)NCCCN(c1cc(C)ccc1)CC	91899375	2.0	-9.3
A26	c1(cc([nH]c1)C(=O)N[C@H](c1ccccc1)CO)c1c(cccc1)CNc1c(cnc(C)c1)C	70701840	2.0	-9.6
A27	c1(cc([nH]c1)C(=O)N[C@H](c1ccccc1)CO)c1c(C)cnc(n1)Nn1c2ccc(Cl)cc2nc1	10252389	2.0	-9.2
A28	c1([nH]cc(c1)c1c(C)cnc(Nc2ccccc2)n1)C(=O)N[C@@H](Nc1ccc(cc1)OCC)CO	91899407	2.0	-8.1
A29	c1(cc([nH]c1)C(=O)Nc1cc(ccc1)C#C[C@@H](C)CO)c1nc(NOCCC)ncc1C	60090601	3.0	-8.6
A30	C[C@@H]1[C@H]2NC[C@H](NC[C@H]2[C@@H](N)N=C(N[C@@H](C)c2ccccc2)NC1)C(=O)N=C(c1ccccc1)CO	70701676	3.0	-9.3
A31	c1(cc([nH]c1)C(=O)N[C@H](c1[nH]cc(n1)C#N)CO)c1c(C)cnc(n1)Nc1ccccc1	91899271	3.0	-8.4
A32	c1([nH]cc(c1)c1c(C)cnc(Nc2ccccc2)n1)C(=O)N[C@@H](Nc1ccc(cc1)C#C)CO	44481491	3.0	-9.2
A33	c1([nH]cc(c1)c1c(C)cnc(Nc2c(C)c(C)ccc2)n1)C(=O)NCN(c1cc(ccc1)C#C)CO	91899307	3.0	-8.5
A34	c1([nH]cc(c1)c1c(C)cnc(Nc2ccccc2)n1)C(=O)N[C@@H](Nc1ccc(cc1)OCCC)CO	70701734	3.0	-9.6
A35	c1([nH]cc(c1)c1c(C)cnc(Nc2c(C)c(C)ccc2)n1)C(=O)N[C@@H](Nc1ccc(cc1)OCC)CO	91899322	3.0	-9.9
A36	c1([nH]cc(c1)c1c(C)cnc(Nc2c(C)c(C)ccc2)n1)C(=O)N[C@@H](NCc1ccc(cc1)OC)CO	70701760	3.0	-9.9
A37	c1([nH]cc(c1)c1c(C)cnc(Nc2c(C)cc(cc2)N)n1)C(=O)N[C@@H](Nc1ccc(cc1)OCC)CO	70701764	3.0	-9.3
A38	c1([nH]cc(c1)c1c(C)cnc(Nc2ccccc2)n1)C(=O)N[S@@H](Nc1c(C)cc(cc1)Br)CO	91899326	3.0	-9.7
A39	c1(cc([nH]c1)C(=O)N[C@@H](F)c1c(ccc(F)c1)CO)[C@H]1N=C(N[C@@H](CC)CO)NC=C1F	91899327	3.0	-9

A40	c1(cc([nH]c1)C(=O)N[C@H](c1[nH]cc(n1)CCN)CO)c1c(C)cnc(n1)Nc1ccccc1	70701786	3.0	-9
A41	c1(cc([nH]c1)C(=O)N[C@H](c1ccccc1)CO)c1c(cccc1)COc1c(cnc(C)c1)C	70701789	3.0	-9.3
A42	c1(cc([nH]c1)C(=O)NSc1c(c(Cl)cc(Cl)c1)CO)c1c(C)cnc(n1)Nc1ccccc1	44482307	3.0	-8.9
A43	c1(cc([nH]c1)C(=O)Nc1c(ccc(c1)CO)NC(=N)N)c1nc(Nc2c(c(ccc2)OC)OC)ncc1C	91899381	3.0	-9.5
A44	c1([nH]cc(c1)c1c(C)cnc(Nc2c(c(ccc2)OC)OC)n1)C(=O)NNNc1cc(ccc1CO)OC	91899384	3.0	-8.8
A45	c1(cc([nH]c1)C(=O)N[C@H](c1ccccc1)CO)c1c(cccc1)OCc1c(C)cnc(n1)Cl	91899389	3.0	-9.4
A46	c1(cc([nH]c1)C(=O)N[C@H](C(=O)c1[nH]ccn1)CO)c1c(C)cnc(n1)Nc1ccccc1	91899403	3.0	-8.3
A47	c1(cc([nH]c1)C(=O)N[C@H](c1ccccc1)CO)[C@H]1c2c(C(=NC[C@@H]1C)NCCC)cccc2	70701876	3.0	-8.5
A48	c1(cc([nH]c1)C(=O)NSNc1cc(ccc1)C(=O)CO)[C@H]1N=C(N[C@@H](CC)CO)NC=C1F	44480711	4.0	-9.3
A49	C1(=CCC[C@@H]2C(=NC[C@H](C)[C@H](NN2)c2cc([nH]c2)C(=O)N[C@H](c2ccccc2)CO)C=C1)NC(=O)C	91899263	4.0	-9.5
A50	c1([nH]cc(c1)c1c(C)cnc(Nc2ccccc2)n1)C(=O)N[C@@H](NOCc1ccc(cc1)OC)CO	91899289	4.0	-8.6
A51	c1(cc([nH]c1)C(=O)NNCc1ccc(cc1)O[C@@H](C)CO)c1c(C)cnc(n1)Nc1ccccc1	44481492	4.0	-10
A52	c1(cc([nH]c1)C(=O)N[C@H](c1ccccc1)CO)c1c(cccc1)OCc1c(C)cnc(c1)Br	91899301	4.0	-9.4
A53	c1(cc([nH]c1)C(=O)N[C@H](c1ccccc1)CO)c1c(C)cnc(n1)c1c2[nH]c(=S)ccn2cn1	91899314	4.0	-10
A54	c1([nH]cc(c1)c1c(C)cnc(Nc2c(C)c(C)ccc2)n1)C(=O)Nc1ccc(cc1)N(CC#C)CO	91899323	4.0	-8.1

A55	c1(cc([nH]c1)C(=O)NNc1ccc(cc1)NC(=O)CO)c1nc(Nc2c(C)cc(cc2)N)ncc1C	70701762	4.0	-9.5
A56	c1(cc([nH]c1)C(=O)/N=C(/c1cc(Cl)c(cc1)OBr)\CO)c1c(C)cnc(n1)Nc1ccccc1	91899352	4.0	-8.2
A57	c1(cc([nH]c1)C(=O)N[S@H](c1ccc(cc1)C#N)CO)c1nc(Nc2c(c(ccc2)OC)OC)ncc1C	70701811	4.0	-9.5
A58	c1(cc([nH]c1)C(=O)N[C@H](c1ccccc1)CO)c1c(C)cnc(n1)n1c(=O)n(cc1)CC(=O)O	91899365	4.0	-9
A59	c1(cc([nH]c1)C(=O)N[C@H](c1ccccc1)CO)c1c(C)cnc(n1)n1c(=S)n(cc1)CC(=O)O	91899366	4.0	-8.6
A60	c1(cc([nH]c1)C(=O)Nc1c(ccc(c1)CO)NC(=N)N)c1c(C)cnc(n1)NN1CCOCC1	44482036	4.0	-9.4
A61	c1(cc([nH]c1)C(=O)N[C@H](c1ccccc1)CO)[C@@H]1C[C@H](CC1)Nc1nc(C)ncc1C	91899374	4.0	-9
A62	c1(cc([nH]c1)C(=O)Nc1c(c(C)ccc1)NC(=O)CO)[C@H]1N=C(N[C@@H](CC)CO)NC=C1F	70701832	4.0	-9.4
A63	c1(cc([nH]c1)C(=O)Nc1c(ccc(c1)CO)NC(=N)N)c1nc(Nc2nc(C)cs2)ncc1C	91899380	4.0	-9.4
A64	c1([nH]cc(c1)c1c(C)cnc(Nc2c(c(ccc2)OC)OC)n1)C(=O)NOc1c(ccc(c1)CO)OCCC=C	70701842	4.0	-9.1
A65	c1(cc([nH]c1)C(=O)N[C@H](c1ccccc1)CO)[C@@H]1[C@@H](C)C=NC(=O)N(C1)c1ccccc1	70701856	4.0	-8.2
A66	c1(cc([nH]c1)C(=O)N[C@H](c1ccccc1)CO)[C@@H]1[C@@H](C)C=NC(=O)N[C@H]1c1ccccc1	70701877	4.0	-8.6
A67	c1(cc([nH]c1)C(=O)N[C@H](c1ccccc1)CO)C1=C[C@H](C(=O)N=C[C@@H]1C)c1ccccc1	70701881	4.0	-8.5
A68	c1(cc([nH]c1)C(=O)Nc1c(cc(cc1)CO)NC(=N)N)c1nc(N[C@@H](CC)COC)ncc1C	91899421	4.0	-8

A69	CC1=CNC2=NC[C@H](CCC[C@@H]2CN[C@@H]1c1cc([nH]c1)C(=O)N[C@H](c1ccccc1)CO)C(=O)NN	70701677	5.0	-9.1
A70	c1([nH]cc(c1)c1c(C)cnc(Nc2c(C)cc(cc2)N)n1)C(=O)N[S@@H](Nc1ccc(cc1)CCC)CO	91899272	5.0	-8.9
A71	c1(cc([nH]c1)C(=O)N[C@H](c1ccccc1)CO)C1=C(CN=C(NC)NC(=N1)NC1CCC1)C	70701718	5.0	-8.3
A72	c1(cc([nH]c1)C(=O)Nc1[nH]c(cn1)C(=O)CO)c1c(C)cnc(n1)Nc1c(C)c(C)ccc1	70701755	5.0	-7.7
A73	Clc1cc(ccc1)[C@H](NC(=O)c1[nH]cc(c1)-c1nc(ncc1F)N[C@@H](CC)CO)CO	70701801	5.0	-8.9
A74	O1C[C@@H](Nc2nc(-c3cc(n(c3)C)C(=O)N[C@H](CO)c3ccccc3)c(cn2)C)CC1	91899355	5.0	-9.1
A75	Clc1cnc(nc1-c1cc([nH]c1)C(=O)N[C@H](CO)c1cc(ccc1)C)NOCC	44482041	5.0	-9.6
A76	Clc1cc(ccc1)[C@H](N(C(=O)c1[nH]cc(c1)-c1nc(ncc1C)N[C@@H](CC)CO)C)CO	44482303	5.0	-9.5
A77	Clc1cnc(nc1-c1cc(n(c1)C)C(=O)N[C@H](CO)c1ccccc1)N[C@@H](CC)CO	70701857	5.0	-8.7
A78	Clc1cc(ccc1)[C@H](NC(=O)c1[nH]cc(c1)-c1nc(ncc1C)N[C@@H](CC)CO)COP(=O)([O-])[O-]	70701868	5.0	-8.1

Table S2. Pharmacophoric features of Ek1, Ek2, Ek3, Ek3, and PubChem91899270

Molecules	HBA	HBD	HY	RA
Ek1	3	3	5	0
Ek2	3	3	3	4
Ek3	3	3	5	4
Ek4	4	3	5	4

PubChem91899270	4	3	5	4

HBA: Hydrogen bond acceptor; HBD: Hydrogen bond donor; HY: Hydrophobic, RA: Ring aromatic

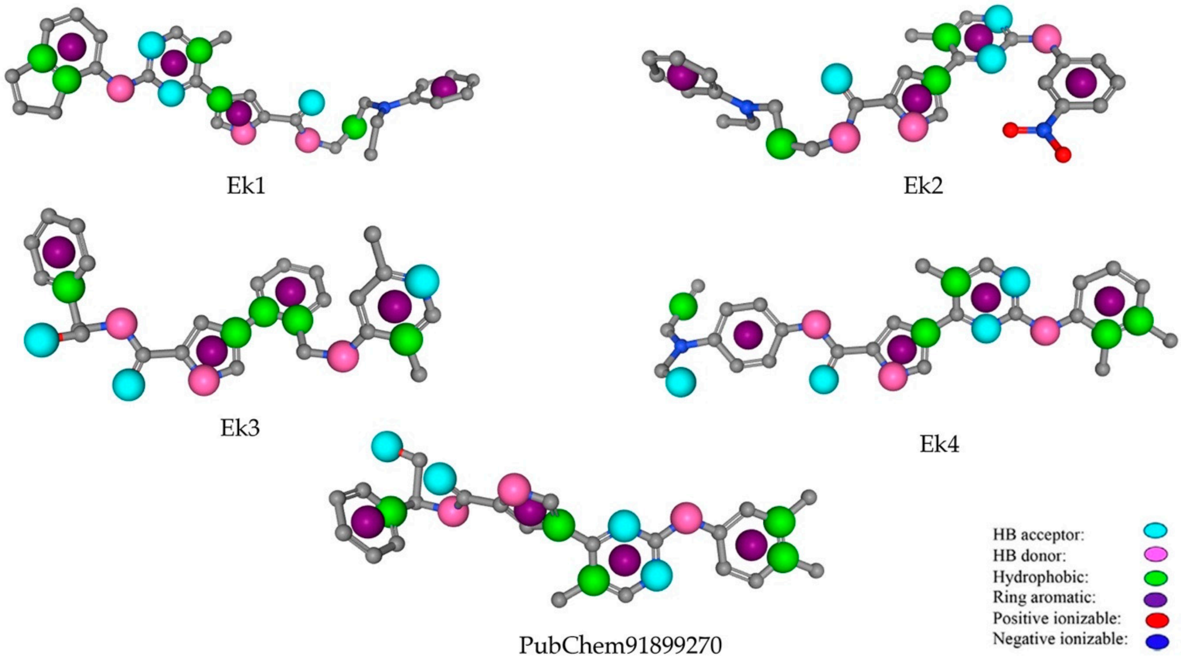

Figure S1. Pharmacophoric features of Ek1, Ek2, Ek3, Ek3, and PubChem91899270

Table S3. Binding affinity and ADMET parameters of ERK2 inhibitors

Parameters	LY3214996	BVD-523	MK-8353
AutoDock Vina binding affinity (Kcal/mol)	-8.72	-8.91	-9.12
K_{DEEP} binding affinity (Kcal/mol)	-5.028	-5.268	-7.902
DiffDock confidence score (Kcal/mol)	-0.77	-2.13	-1.83
Molecular weight	453.572	433.339	691.862
Gastrointestinal permeability	94.918	89.613	86.768
Skin permeability	-2.736	-2.762	-2.735
Synthetic accessibility	4.01	3.59	5.58

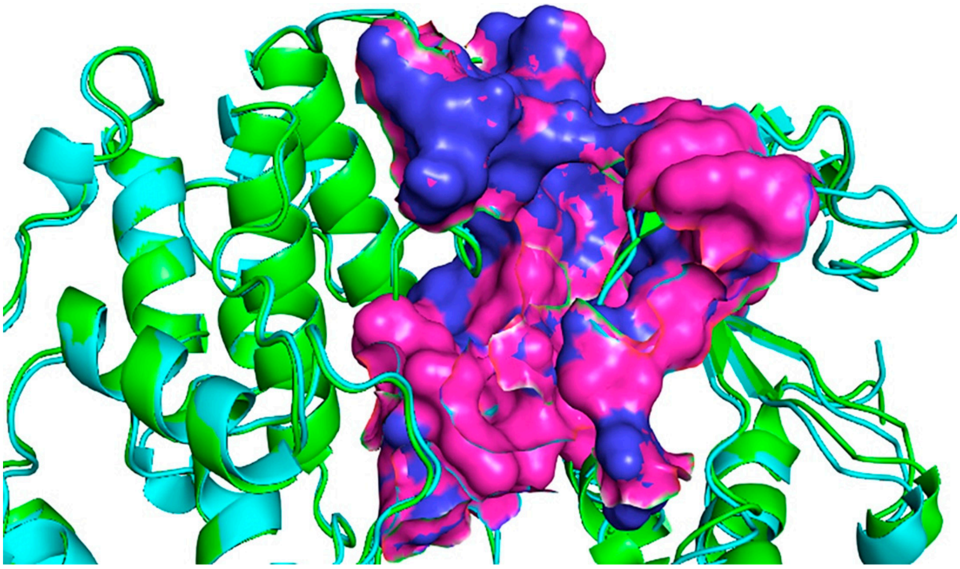

Figure S2. Superimposition of ERK2 and ERK1 active sites obtained from the ProBis server.
Blue: ERK2; Pink: ERK1
